# Effect of Pulse Current and Pre-annealing on Thermal Extrusion of Cu in Through-Silicon via (TSV)

**DOI:** 10.3389/fchem.2020.00771

**Published:** 2020-10-14

**Authors:** Youjung Kim, Sanghyun Jin, Kimoon Park, Jinhyun Lee, Jae-Hong Lim, Bongyoung Yoo

**Affiliations:** ^1^Department of Materials Science and Chemical Engineering, Hanyang University, Ansan, South Korea; ^2^Department of Material Engineering, Hanyang University, Ansan, South Korea; ^3^Department of Advanced Material Science Engineering, Hanyang University, Ansan, South Korea; ^4^Department of Materials Science and Engineering, Gachon University, Seongnam-Si, South Korea

**Keywords:** through-silicon-via (TSV), pulse current, pre-annealing, thermal extrusion, seed layer

## Abstract

Thermal stress induced by annealing the Cu filling of through-silicon vias (TSVs) requires further investigation as it can inhibit the performance of semiconductor devices. This study reports the filling behavior of TSVs prepared using direct current and pulse current Cu electrodeposition with and without pre-annealing. The thermal extrusion of Cu inside the TSVs was studied by observing the extrusion behavior after annealing and the changes in grain orientation using scanning electron microscopy and electron backscatter diffraction. The bottom-up filling ratio achieved by the direct current approach decreased because the current was used both to fill the TSV and to grow bump defects on the top surface of the wafer. In contrast, pulse current electrodeposition yielded an improved TSV bottom-up filling ratio and no bump defects, which is attributable to strong suppression and thin diffusion layer. Moreover, Cu deposited with a pulse current exhibited lesser thermal extrusion, which was attributed to the formation of nanotwins and a change in the grain orientation from random to (101). Based on the results, thermal extrusion of the total area of the TSVs could be obtained by pulse current electrodeposition with pre-annealing.

## Introduction

The through-silicon via (TSV) is an important technology for connecting dies in 3D interconnects to overcome the physical and economic limitations associated with wiring and enhance the performance of semiconductor devices (Beica et al., 2008; Motoyoshi, 2009; Cao et al., 2013; Pan et al., 2018). In general, Cu with its high electrical conductivity is used to fill TSVs by deposition methods, such as chemical vapor deposition, physical vapor deposition, and electrodeposition. Among these, electrodeposition is a simple and efficient approach for the cost-effective production of void-free fillings (El-Giar et al., 2000).

After TSV filling, Cu is typically subjected to high-temperature processes such as annealing to stabilize its microstructure (Yang et al., 2011). However, annealing induces thermal stress, which can cause reliability problems such as degraded device performance in keep-out zone and thermal extrusion of Cu (De Wolf et al., 2011; Heryanto et al., 2012; Ryu et al., 2012; Guo et al., 2013). A keep-out zone, or keep-away zone, is an area where the transistor is affected by thermal stress from the mismatched coefficients of thermal expansion between Si (2.3 ppm/°C) and Cu (16.7 ppm/°C); this stress can decrease the carrier mobility and device performance (Selvanayagam et al., 2009; De Wolf et al., 2011; Farooq et al., 2011; Heryanto et al., 2012; Ryu et al., 2012). Cu extruded from the surface of the Si wafer by annealing can destroy the insulating layer or interconnection layer. The stress distribution of a TSV affects the size and performance of the chip because the TSV is larger than the transistor (Chen et al., 2012). In addition, when a TSV, which connects chips and chips, is arranged in multiple arrays, the higher TSV density can cause greater effects from thermal stress during annealing. Therefore, as the dimensions of TSVs continue to decrease, TSV thermal stress becomes a more important issue.

Nanotwins, which can be formed with a pulse current, change the microstructure of Cu and enhance its mechanical properties (Liao et al., 2013; Seo et al., 2014). Lu et al. succeeded in forming nanotwin Cu using a pulse current with a high current density at a low temperature (Lu et al., 2004). Xu et al. formed nanotwin Cu thin films using a pulse current and measured the stress of Cu using an *in situ* measurement system; they found that the nanotwin structure formed due to stress changes induced by repeated pulse on-time and off-time cycles (Xu et al., 2007, 2009). These previous studies reported the relationship between nanotwins and enhanced mechanical properties of Cu thin films. However, there have been no studies on the properties of Cu inside TSVs fabricated by pulse current electrodeposition. In addition, Jing et al. performed a reliability assessment of Cu extrusions in TSVs with controlled annealing conditions including pre-chemical-mechanical polishing (CMP) (Jing et al., 2015). The effect of pre-CMP was also studied by De Wol et al. who showed that the pre-CMP time and temperature after sintering reduced the Cu extrusion (De Wolf et al., 2011). Jing et al. also measured silicon stress and Cu extrusion with different annealing temperatures and pre-annealing by simulation and micro-Raman microscopy (Jing et al., 2014). However, the effect of both electrodeposition conditions and the employment of a pre-annealing process on the crystal structure and mechanical process of Cu in TSVs has not yet been reported.

Chen et al. investigated the effects of small grain size on reducing extrusion and suggested higher current density, higher additive concentration, and optimized annealing temperature ramp rate could control grain size and reduce extrusion (Chen et al., 2016, 2017). The microstructure of grain size was also observed by An et al., who proposed diffusion creep rate model of TSV-Cu (An et al., 2018). The creep deformation caused Cu extrusion through the diffusion of grain boundaries. In addition, Si anisotropy and Cu plasticity on interface cracking has been found to affect extrusion (Dai et al., 2019).

High mechanical strength and electrochemical behaviors are essential characteristics for Cu in TSV to improve the performance of devices. In this study, a nanotwin structure was formed by pulse current to enhance the properties of electrodeposited Cu. In addition, the influence of plating and annealing conditions on the crystal and mechanical properties of Cu in TSVs and changes in the thermal extrusion behaviors were investigated. TSVs were filled with Cu by direct current and pulse current electrodeposition for different processing times as well as with and without pre-annealing. The mechanical properties, morphologies, and thermal extrusion behaviors were measured experimentally, and via filling mechanisms for direct and pulse current deposition were proposed.

## Materials and Methods

**Figure 1:**
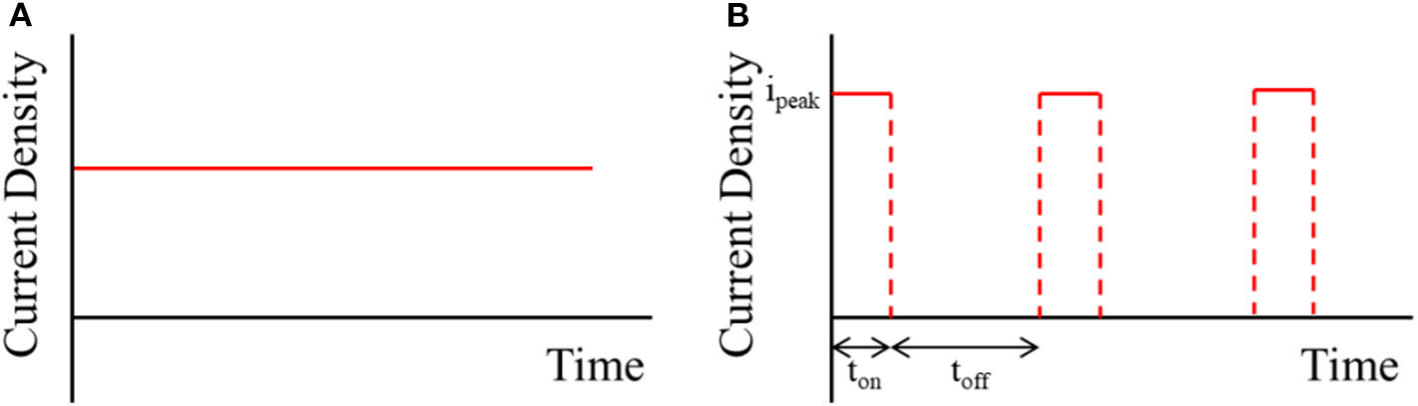
The schematic diagram (current vs. time) of **(A)** direct current and **(B)** pulse current.

The electrolytes for Cu TSV filling were prepared by dissolving 1 M CuSO_4_ (Yakuri Pure Chemicals, 99.5%, Japan) and 1.9 mM HCl (Daejung Chemicals & Metals, 60%, Korea) in deionized water. The pH of the electrolytes was adjusted to 0.5 using H_2_SO_4_ (Junsei Chemical, 95%, Japan). For void-free TSV filling, 100 ppm of suppressor (CuSupH-1, Hangaram Chemistry Co., Korea) was added. All of the Cu filling experiments were conducted without agitation in a 100-mL electrochemical cell with a typical three-electrode system consisting of a Pt-coated Ti plate and Ag/AgCl electrode (Thermo Fisher Scientific, USA) as the counter and reference electrode, respectively. A via-patterned Si wafer with an aspect ratio of 12 (diameter: 5 μm, depth: 60 μm) and SiO_2_/Ta barrier layer/Cu seed layer were utilized as the working electrode. The Cu TSV filling was performed with direct current and pulse current deposition at current densities of 1, 3, 5, and 10 mA/cm^2^ at room temperature using a potentiostat/galvanostat (Princeton Applied Research, VersaSTAT4, AMETEK, Inc., USA) after a diffusion time of 60 s. The filling shapes of the direct current and pulse current are shown in Figures 1A,B. The pulse current comprised various factors, such as peak current density (*i*_peak_), on-time (*t*_on_), and off-time (*t*_off_). The average current density (*i*_avg_) of the pulse current was calculated by the following equation:

(1) $$\begin{array}{r} i_{avg}\;=\frac{i_{peak}\;\times \;t_{on}}{t_{on}\;+t_{off}}. \end{array}$$

In our previous study, the high peak current density and short on-time of the pulse current were optimized with a high frequency of the pulse current. For pulse current deposition, the average current density was controlled by the off-time, and the peak current and on-time were fixed at 450 mA/cm^2^ and 2 μs, respectively.

After the Cu filling, the samples were molded with epoxy resin and polished using a polishing machine (SSAUL BESTECH, BESTPOL P362, Korea) to analyze the filling behavior in the TSVs. The cross-sections of the Cu-filled TSVs were observed using optical microscopy (Hirox, KH-7700, Japan). In addition, the Cu films electrodeposited with both direct current and pulse current on nonpattern wafers with a Ti layer were used for tensile stress–strain testing. The Cu film of 10 μm was electrodeposited with an applied current time of 226 min. The tensile strength of the deposited Cu was measured by a universal testing machine (UTM, INSTRON, model 3343, USA).

**Figure 2:**
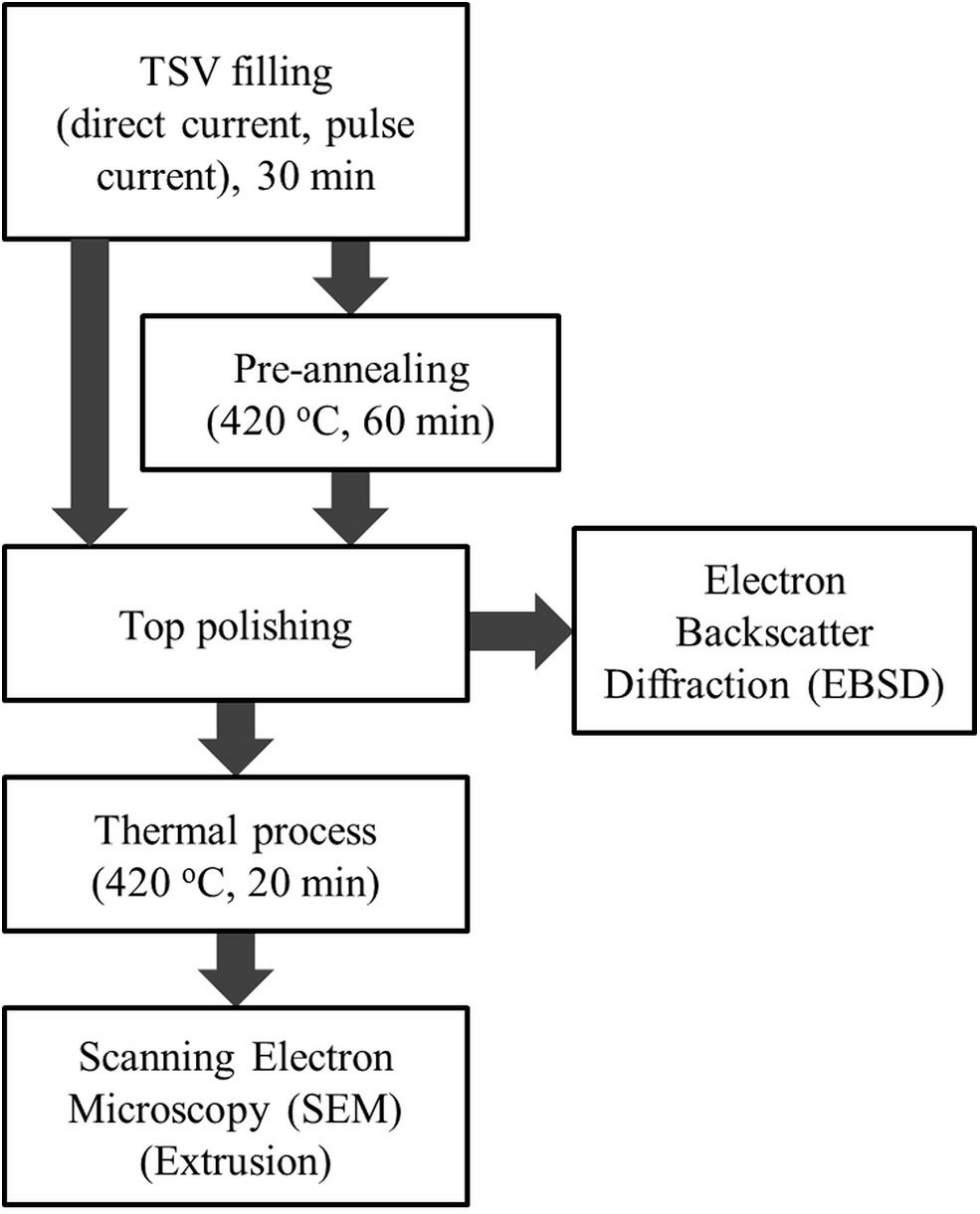
Process flow of thermal annealing.

To investigate the effects of Cu thermal extrusion in the TSV, the TSVs were fully filled and mechanically polished to expose the top surface of the via using a polishing machine. Figure 2 shows the experimental procedure. The prepared samples were pre-annealed and annealed at 420°C for 60 and 20 min, respectively, using rapid thermal annealing under an N_2_ atmosphere after TSV filling. The ramp-up and ramp-down rate were set as 84°C/min and 7°C/min, respectively. The distributions of Cu grain orientation angles in the TSVs depending on current type (direct or pulse) and the use of pre-annealing were observed by electron backscatter diffraction (EBSD, TESCAN, MIRA3) after top polishing. Annealing was performed after top polishing under an atmosphere of 95% Ar and 5% H_2_ to prevent oxidation. The thermally extruded top surfaces of the TSVs were observed by field emission scanning electron microscopy (FE-SEM, TESCAN, model MIRA3) for the selected current types (direct and pulse) and with and without pre-annealing.

## Results and Discussion

### TSV Filling Using Pulse Current

**Figure 3:**
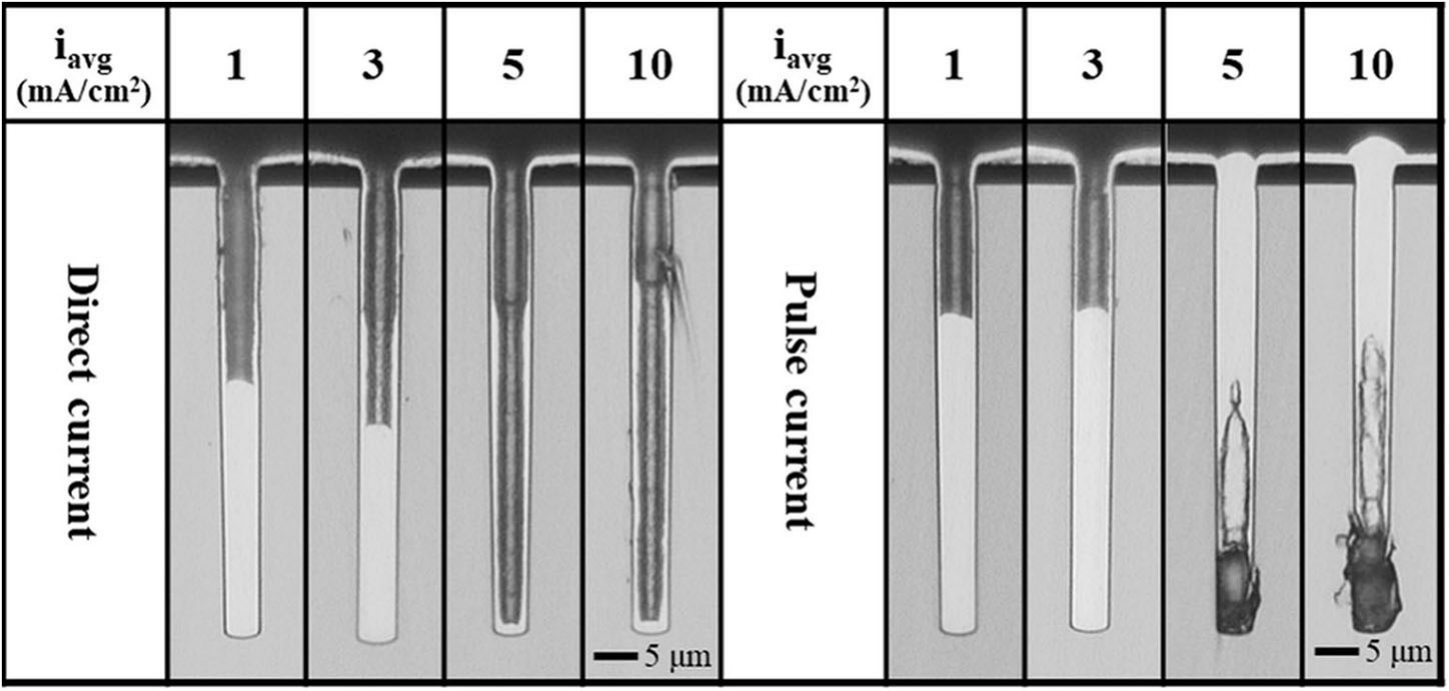
Cross-sectional images of Cu-filled TSVs deposited by direct current and pulse current for 10 min.

The cross-sectional optical images of Cu-filled TSVs deposited for duration of 10 min with direct and pulse currents are shown in Figure 3. In pulse current electrodeposition, the on-time and frequency are critical factors for TSV filling. The effect of on-time on TSV filling was investigated in our previous study (Jin et al., 2013). In that study, on-time and frequency affected the coulombic current density and dissolution rate, and the fastest void-free bottom-up filling was achieved with an on-time of 2 μs. Therefore, for pulse current electrodeposition, the peak current density and on-time were fixed at 450 mA/cm^2^ and 2 μs, respectively. The TSV filling ratio using a direct current decreased with increasing average current density because of the current lost to the additional growth of bump defects on the surface (Supplementary Figure 1). The filling ratio was calculated as the height of the filled Cu relative to the depth of the TSV. Electrodeposition using direct and pulse currents had the highest filling ratios without bump defects at 1 and 3 mA/cm^2^, respectively (number of bump defects shown in Supplementary Figure 1). During direct current electrodeposition, the suppressor on the top surface of the wafer desorbed, and thus bump defects formed in the suppressor desorbed region. Bottom-up filling did not begin with 5 or 10 mA/cm^2^ because many bump defects formed. In contrast, the suppressor is re-supplied during the off-time using a pulse current. Therefore, the filling ratio for pulse current electrodeposition was higher than that of direct current deposition because the top surface of the wafer was strongly suppressed and thus the current was only used for TSV filling instead of defect formation. However, the supply of suppressor in the TSV was not sufficient for deposition above an average current density of 5 mA/cm^2^ due to the short off-time. In addition, the suppressor easily desorbed from the sidewall of the TSV under very high current densities using pulse current electrodeposition, resulting in a pinch-off effect at the sidewall and a void because of the much higher current density and lower suppressor concentration (Song et al., 2012; Wheeler et al., 2013; Yang et al., 2013; Wang et al., 2016, 2018; Xiao et al., 2017).

**Figure 4:**
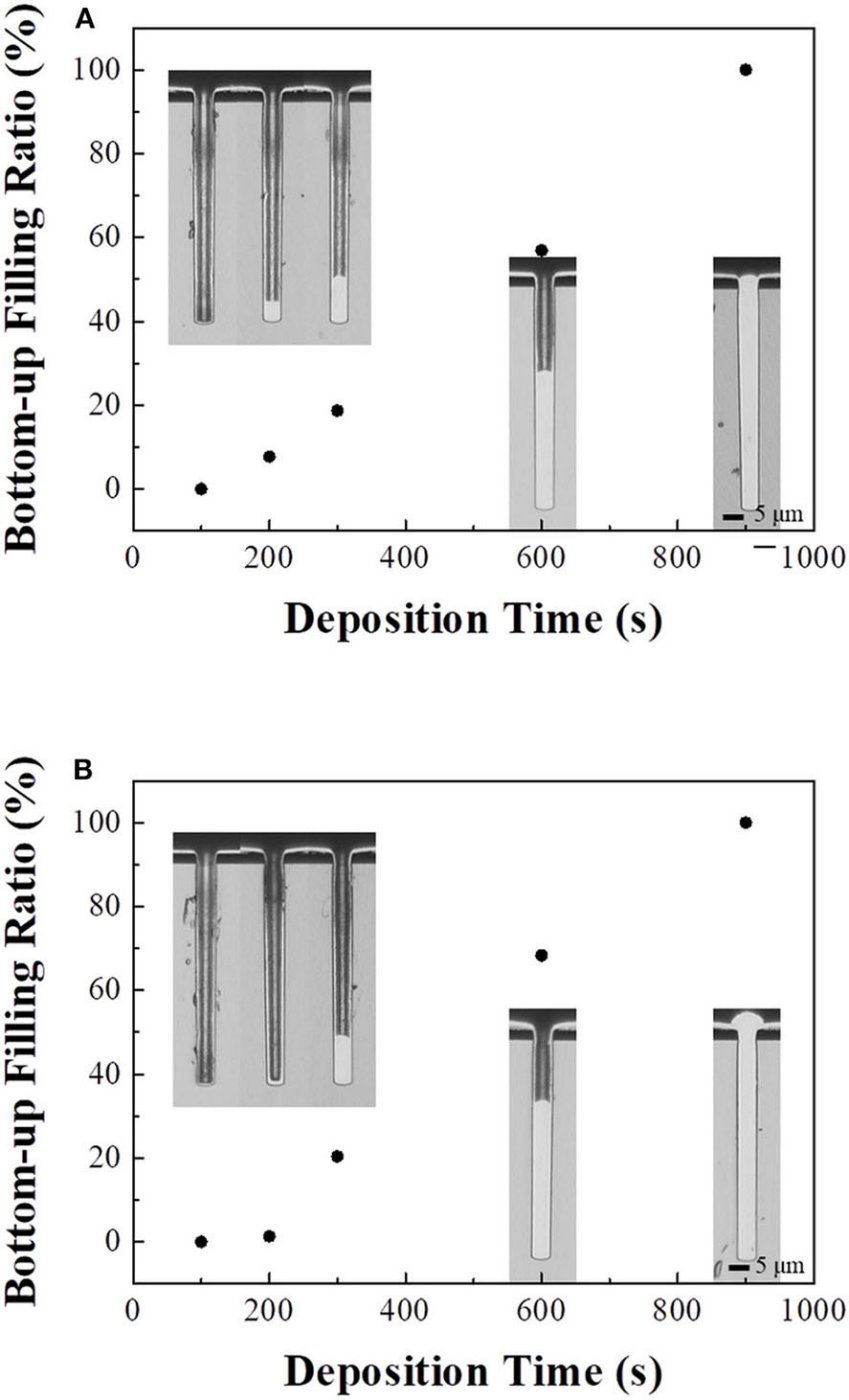
Cross-sectional images of Cu-filled TSVs and their bottom-up filling ratios depending on deposition time. **(A)** Direct current (1 mA/cm^2^), **(B)** pulse current (3 mA/cm^2^).

Cross-sectional images and bottom-up filling ratios until filling completion acquired with the best defect-free conditions (1 and 3 mA/cm^2^ for direct and pulse currents, respectively) for each deposition time are shown in Figure 4, with the results for direct current electrodeposition in Figure 4A. The initial filling using direct current electrodeposition reached completion in less than 200 s, and the filling ratio linearly increased because the current was effectively used for TSV filling. In addition, the linear slope of the filling ratio with respect to deposition time from the start might indicate a constant thickness of the diffusion layer of cupric ions and a sufficient ion supply. However, it may be difficult for the cupric ions to diffuse into the via for a TSV with a high-aspect ratio (Gambino et al., 2015; Lee and Chen, 2018). In addition, as shown in Figure 4B, the bottom-up filling ratio using pulse current electrodeposition was lower than that using a direct current at the initial stage due to re-adsorption of the suppressor during the off-time. The adsorbed suppressor could interrupt the initial bottom-up filling; the bottom-up filling then began after the breakdown of the suppressor on the bottom of the via (Wheeler et al., 2013; Yang et al., 2013). However, the slope of the pulse current filling ratio with respect to deposition time becomes linear and steeper than that of the direct current from 200 s onward, indicating a shorter diffusion layer and higher filling ratio. The diffusion layer using a pulse current was reduced because of the supply of cupric ions provided during the off-time (Chandrasekar and Pushpavanam, 2008).

**Figure 5:**
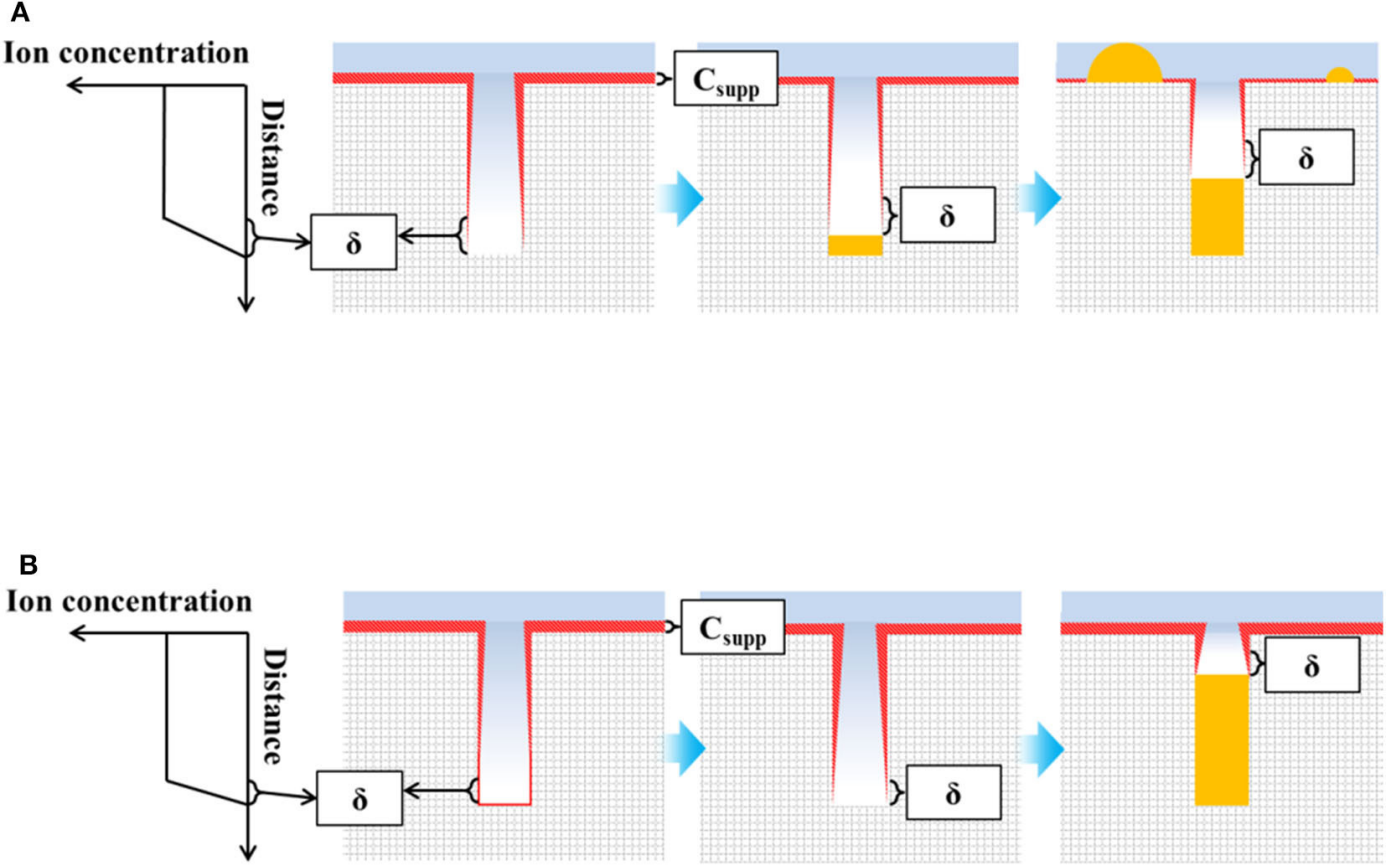
Schematic of suppressor and ion concentration for TSV filling and filling mechanisms with a **(A)** direct current and **(B)** pulse current (δ is the diffusion layer; the thickness of C_supp_ indicates the suppressor concentration).

The proposed TSV filling mechanisms for direct current and pulse current electrodeposition are shown in Figure 5. The concentrations of cupric ions and suppressor decrease gradually toward the bottom of the via before electrodeposition. Figure 5A shows the filling mechanism using a direct current. The suppressor at the bottom of the TSV easily desorbs by breaking down, and the TSV filling begins from the bottom when the current is initially applied. The filling ratio remains constant over time because of the sufficient diffusion of cupric ions to the TSV. However, the suppressor on the wafer surface also desorbs, resulting in bump defects and thus a lower filling ratio. However, the sufficient supply of cupric ions and suppressor using pulse current electrodeposition results in a shorter diffusion layer of cupric ions and increased adsorption of the suppressor, as shown in Figure 5B. Therefore, bottom-up filling does not begin by adsorption of the suppressor on the via bottom. Instead, TSV filling starts after desorption of the suppressor on the bottom of the TSV with a higher filling ratio due to the short diffusion layer. In addition, unlike direct current deposition, pulse current deposition does not form bump defects on the surface because the suppressor is re-adsorbed during the off-time. In other words, the high filling ratio using pulse current deposition was attributed to the shorter diffusion layer and lack of bump defect formation.

### Mechanical Properties Using Pulse Current

**Figure 6:**
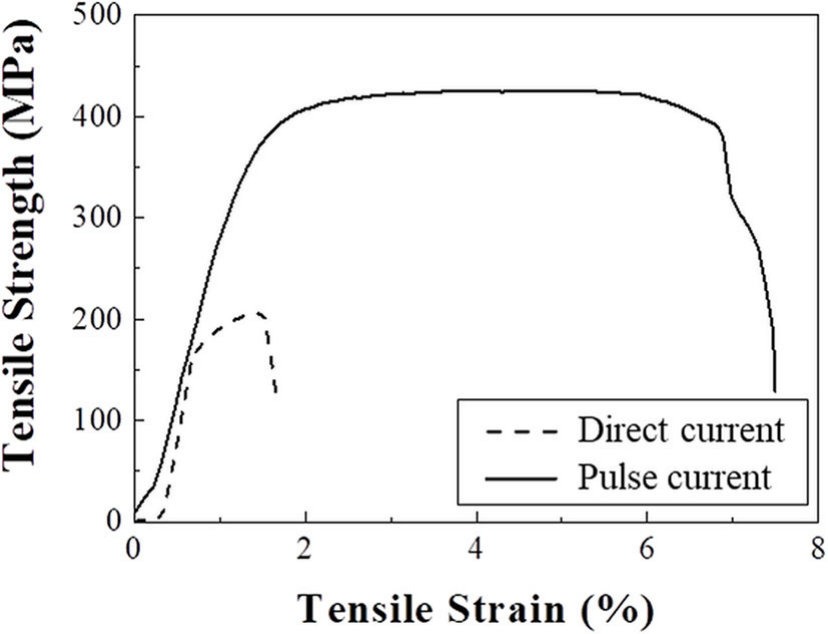
Tensile stress–strain curves of samples electrodeposited with a direct current and a pulse current.

In addition to increasing the TSV filling ratio, pulse current deposition also forms a nanotwin structure. In our previous work, a high density of nanoscale twins was observed under pulse current conditions (Jin et al., 2013). In addition, the thermal extrusion behavior, which is related to mechanical strength, decreased with nanotwin structures. Lu et al. similarly concluded that nanotwins could increase mechanical strength (Lu et al., 2004). Therefore, stress–strain curves were measured to investigate the mechanical strength of Cu plated with direct and pulse currents. Figure 6 shows the stress–strain curves of samples electrodeposited with a direct current and a pulse current with an. Stress–strain curves consist of elastic and plastic regions (Sharir et al., 2008). In the elastic region, which has a linear slope before the yield strength, only elastic (reversible) deformation occurs, whereas non-reversible (permanent) deformation begins to occur in the plastic region located between the yield strength and fracture point. Therefore, the yield strength can indicate the maximum allowable strength before plastic deformation occurs (Yonenaga, 2005). The 0.2% offset strength, which is defined as the stress that results in a strain of 0.2%, was measured for engineering evaluation instead of yield strength (Albrecht et al., 2017). The Cu formed by a direct current had a 0.2% offset strength of 186 MPa, which resulted in plastic deformation at a low strain energy, while the Cu formed by a pulse current had a higher 0.2% offset strength of 372 MPa, allowing it to deform elastically without plastic deformation at a higher strain energy. The higher offset strength was due to the nanotwin boundaries interrupting the migration of dislocations (Lu et al., 2004; Jin et al., 2013). Grain boundaries also block the migration of electrons, resulting in a high electrical resistance. However, nanotwin boundaries block the migration of dislocations without disturbing the transport of electrons. Therefore, the twin boundaries formed by a pulse current can increase the mechanical strength of Cu without sacrificing electrical conductivity.

### Thermal Extrusion of TSV Using Pulse Current and Pre-annealing

**Figure 7:**
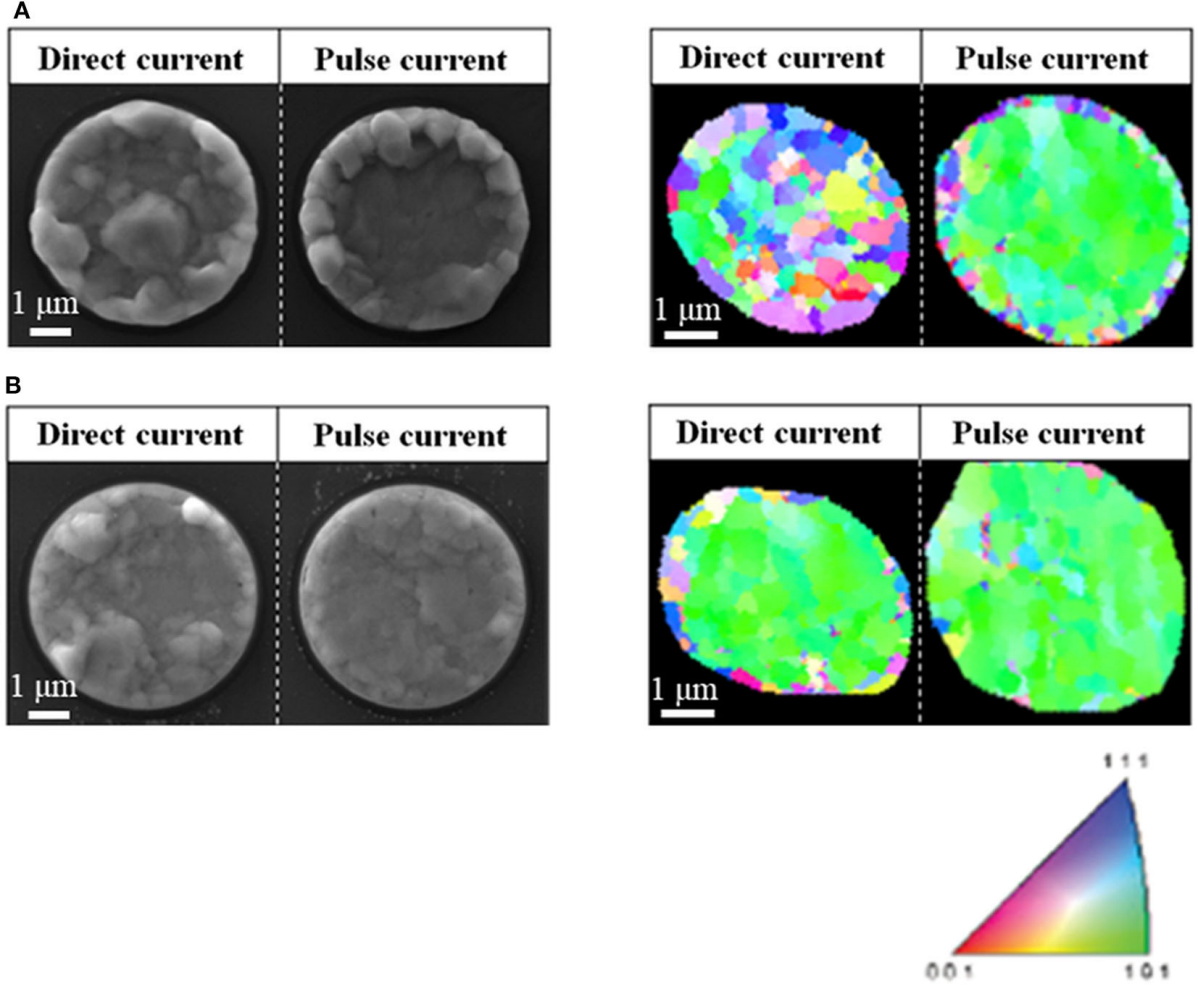
Thermal extrusion behaviors and grain orientations (EBSD analysis) of TSVs depending on current type (direct current 1 mA/cm^2^, pulse current 3 mA/cm^2^) **(A)** before and **(B)** after pre-annealing.

The thermal extrusion behaviors and EBSD analysis of TSVs prepared with direct current and pulse current plating are illustrated in Figure 7A. The thermal extrusion behavior was observed after annealing at 420°C for 20 min in an N_2_ gas atmosphere. The Cu electrodeposited using direct current inside the TSV was severely expanded after annealing, which is attributed to plastic deformation from thermal stress exceeding the yield strength (Heryanto et al., 2012). In contrast, thermal extrusion occurred to a lesser extent in the Cu deposited with a pulse current, which is attributed to the Cu nanotwins allowing only for elastic deformation during annealing. However, the Cu at the edge of the TSV severely expanded even with the use of a pulse current. The proportion of the extruded area among the total TSV area for each direct current and pulse current condition was 71.9 and 47.7%, respectively. EBSD analysis was used to investigate the grain orientation before thermal extrusion. The Cu plated with a direct current had a random orientation, resulting in thermal extrusion throughout, whereas the Cu plated with a pulse current had a (101) orientation except at the TSV edge, resulting in more thermal extrusion at the edge. Thermal extrusion seems to be affected by not only nanotwins but also preferred orientation. The random crystal orientation at the edge might be attributable to the Cu seed layer formed by physical vapor deposition. Thus, further methods such as an additional annealing process are necessary to improve the crystal orientation and minimize thermal extrusion. The thermal extrusion behaviors and EBSD analysis of TSVs prepared with a direct current and pulse current after an additional pre-annealing treatment are shown in Figure 7B. Pre-annealing was carried out at 420°C for 60 min in an N_2_ gas atmosphere. After pre-annealing, the proportion of the extruded area among the total TSV decreased from 71.9 to 47.0% under the direct current condition and from 47.7 to 23.7% under the pulse current condition. The pre-annealing also changed the grain orientation at the center of the Cu plated by a direct current to the (101) orientation and grain orientation at the edge of the Cu plated by a pulse current to the (101) orientation as determined by EBSD. Supplementary Figure 2 shows that the dominant preferred orientation of both the direct current and pulse current Cu samples was further improved toward the (101) orientation after pre-annealing. Therefore, it is important to note that controlling the density of nanotwin boundaries and the grain orientation could improve the mechanical properties of TSVs and reduce the extent of thermal extrusion.

## Conclusions

The filling behaviors and thermal extrusion of the high-aspect ratio Cu in TSV using pulse current with pre-annealing were much improved. In summary, The pulse current plating formed less bump defect on the surface, which indicates low current loss high filling efficiency than direct current one because the cupric ion and suppressor were supplied during off-time. In addition, mechanical properties of Cu electrodeposited using pulse current were improved. The tensile strength of Cu electrodeposited with pulse current was 425 MPa, which was higher than one of direct current (206 MPa). The higher tensile strength and more (101) preferred orientation of Cu using pulse current much reduced thermal extrusion.

## Data Availability Statement

The original contributions presented in the study are included in the article/Supplementary Material, further inquiries can be directed to the corresponding author/s.

## Author Contributions

The concept for this study was designed by BY, SJ, and J-HL. BY and SJ designed the experiment. YK, SJ, and JL performed experiments on electrodeposition and KP helped in stress test. SJ and YK performed experiments on EBSD and analyzed EBSD data. All authors helped to write the manuscript.

## Conflict of Interest

The authors declare that the research was conducted in the absence of any commercial or financial relationships that could be construed as a potential conflict of interest.

## Abbreviations

TSV: through-silicon via
EBSD: electron backscatter diffraction
pre-CMP: pre-chemical–mechanical polishing.
