# Supplementary material for: Effect of Pulse Current and Pre-annealing on Thermal Extrusion of Cu in Through-Silicon via (TSV)
Source: Front Chem. 2020 Oct 14;8:771. doi: 10.3389/fchem.2020.00771 (PMC7591792; doi:10.3389/fchem.2020.00771)
Supplement: Supplementary file 1 [file Data_Sheet_1.docx]

Supplementary Material

# Supplementary Figures


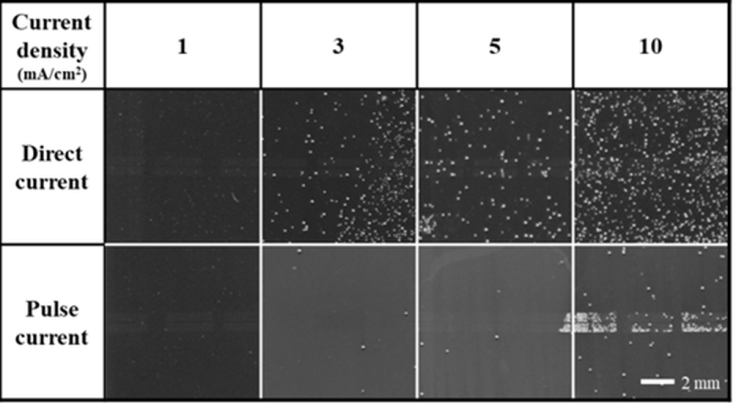


**Supplementary Figure 1.** Top surface morphologies and bump defects of Cu-filled TSVs prepared with a direct current and pulse current for 10 min of processing time.

**
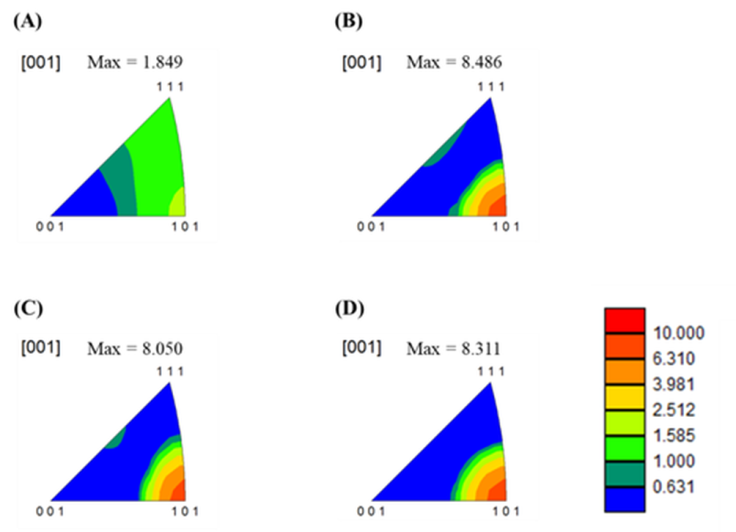
**

**Supplementary Figure 2.** Inverse pole figure intensity distributions of zones of (A) direct current w/o pre-annealing, (B) direct current w/ pre-annealing, (C) pulse current w/o pre-annealing, and (D) pulse current w/ pre-annealing.
